# Effectiveness of medical ethics education: a systematic review

**DOI:** 10.15694/mep.2021.000175.1

**Published:** 2021-06-15

**Authors:** Filipa Moreira, Pedro Teixeira, Cecília Leão

**Affiliations:** 1 Hospital de Braga; 2 School of Medicine

**Keywords:** Medical ethics, Medical education, Medical practice, Ethical training, Ethics competence, Effectiveness, Impact.

## Abstract

This article was migrated. The article was marked as recommended.

Background

Medical ethics is universally accepted as a fundamental part of medical education. One of the current challenges lies in assessing its effectiveness. The primary objective is to analyze the impact of training in medical ethics, and secondly to describe educational practices and discuss the most effective and appropriate pedagogical models.

Methods

The PubMed and EMBASE databases were searched for studies up June 2019.

Studies with a focus on assessing teaching medical ethics were considered. The included population were medical students, residents or faculty physicians with quantitative measured outcomes with at least one of the following criteria: i) pre and post intervention evaluation or ii) a comparison with a control group that did not receive the educational intervention.

Results

A total of 26 studies ranging from 1990 to 2017 were included: 12 (46%) with medical students, 12 (46%) with residents and 2 (8%) with faculty physicians. The most common outcomes are Knowledge, Confidence and Attitudes/ Behaviour. Assessment instruments are knowledge tests, self-assessment questionnaires, reviewing clinical charts and OSCE. Positive statistically significant differences were found in outcomes in 19 (73%) studies.

Conclusions

A great heterogeneity was found in the way of teaching, assessment and measured outcomes. Most studies focus in medical students or residents. Very few studies present follow-up measures, simulation training and validated and standardized assessment tools with behavioural components. Therefore, the evidence to support the positive impact remains weak. Future research on medical ethical training ought to place similar effort and rigour as other clinical competence skills.

## Background

Medical ethics has undergone major changes over the centuries. It has been influenced by religion and social modifications, unprecedented growth in scientific knowledge, expansion in the availability and efficacy of medical technologies, new organizational arrangements in the provision of services and increased pressure to contain spiralling costs. Societal expectations of doctors have also changed profoundly. Patients have much higher expectations of their physicians in terms of safe surgery and personalized care. Moreover, patient’s trust of physicians in this technologically and organizationally complex setting is often low ( Schmitz, Chow and Rothenberger, 2012).

Medical ethics is expected to play an important role in the future, particularly related to: artificial intelligence ( *e.g.* decision algorithms, machine learning, biobanks), genetics ( *e.g.* genetic engineering, reproductive biology and gene therapy), health systems ( *e.g.* development and maturation of medical practice, decision support systems, resource allocation for health policy, planning, finances and human resources), health literacy ( *e.g.* public education about rights and responsibility, concepts of biomedical ethics to avoid ethical conflicts, misunderstanding between patients and physicians) and physician-patient relationship with a gradual shift from traditional paternalism to a patient-centred model ( Nandi, 2000) ( Lehmann, Sulmasy and Desai, 2018).

The need to combine medical innovations with a preservation of human values modifies the role of a physician who must also find out the “right thing to do” in a variety of situations with ethical dilemmas.

Currently there is a wide acceptance that medical ethics is a key feature of a medical curriculum (horizontally and vertically) and requires input from several disciplines and professions ( Campbell, Chin and Voo, 2007).

The underpinnings of Medical ethics education and desired outcomes have been discussed in terms of knowledge, habituation and action. Knowledge and habituation play a role in clinically proper conduct. Habituation must also involve becoming a reflective practitioner; which comprises critical thinking and ethical awareness in regular practice ( Campbell, Chin and Voo, 2007).

Evaluating the impact of medical ethics teaching is a difficult and challenging task. This may be associated with the lack of effective and appropriate pedagogical models with suitable measures for evaluating the impact of continuous ethical training on the medical practice. Regarding knowledge, some of the assessment tools described in the literature include essay-style question and multiple-choice questions. Concerning habituation, the use of case reports (reflecting on ethical circumstances), portfolios’ (recording medical student’s progress) and writing of case vignettes (to test the identification of ethical issues in particular situations). At level of action, OSCEs (Objective Structured Clinical Examination) and feedback from several sources may be the better way of assessing competence ( Campbell, Chin and Voo, 2007).

Medical Ethics is taught and assessed both in qualitative and quantitative measures. Quantitative methods offer several benefits such as: comparing among groups (i.e. pre-test vs. post-test; experimental vs control), or quantifying change on matters that can be attributed to ethics intervention. They are also more prone to replication if procedures are clearly reported and valid standardized instruments employed. Qualitative methods will capture a more holistic perspective and have the potential to present a better understanding into how and why ethics education is effective. Quantitative methods may be helpful for asserting medical ethics training effectiveness ( Eckles *et al,* 2005) ( Watts *et al.,* 2017).

Despite the existing literature on medical ethics education and its attempts to assess effectiveness, profound deficiencies continue to be described about overall goals, teaching methods, outcomes, and assessment tools ( Eckles *et al.,* 2005)( Watts *et al.,* 2017).

This article explores challenges faced by medical ethics education and a critical appraisal of current accomplishments, from the undergraduate, graduate to post-graduate level.

## Objectives

The study primary objective was to evaluate the effectiveness of medical ethics teaching identifying its difficulties and limitations and how does the continuous ethical training affect the medical practice. Secondarily, describe educational practices and discuss the most effective and appropriate pedagogical models.

## Methods

A standardised methodology for conducting educational systematic review was performed ( Hammick, Dornan and Steinert, 2010).

### Criteria for considering studies for this review

Inclusion criteria: i) studies published in English up June 2019 describing an ethics educational intervention, ii) focusing in medical students, residents or faculty physicians, iii) with quantitative outcome measures with at least pre and post interventions measurements or intervention and control groups’ measurements.

Exclusion criteria: i) studies with other health professionals ( *e.g.* nurses, dentists, veterinarians), ii) focusing on medical research rather than clinical practice, iii) focusing on communication skills instead of ethical dilemmas, iv) with qualitative outcome measures and v) opinion or revision papers.

### Types of studies

All studies included are primary studies with an educational intervention. Search found randomized studies, controlled non-randomized studies, retrospective and prospective cohort studies.

### Types of participants

Study participants ranged from medical students, residents ( *viz.* radiology, paediatric, internal medicine, general surgery, obstetrics/gynaecology, orthopaedic and family medicine) to faculty physicians.

### Types of interventions

Educational interventions included one or more of the following: seminars/lectures, workshops, small group discussions ( *e.g.* case-based discussions), case analysis and writing-based teaching and role-play ( *e.g.* standardized patients/students).

In order to simplify the studies analysis interventions were classified in 3 types: type I ( *i.e.* traditional seminar - grand lectures), type II ( *i.e.* case based - small group discussion), type III ( *i.e.* role-play - standardized patients/students).

### Types of outcome

Assessment instruments included knowledge tests ( *i.e.* multiple choice, open-ended question, true-false), self-assessment questionnaires/surveys ( *i.e.* Likert type scales), reviewing clinical charts and OSCEs.

### Primary outcomes

Included studies have as primary outcomes measures: Knowledge, Confidence and Attitudes/ Behaviours.

### Secondary outcomes

One or more of the following secondary outcomes were found: demographic data, history of previous medical ethics courses/education, degree of satisfaction with the intervention performed and personal experiences with ethical dilemmas.

### Search methods for identification of studies

Search was conducted utilizing PubMed and EMBASE databases as well as the reference list of relevant research publications and reviews. Combinations of the following key terms were employed within the literature search: ethics, bioethics, medical ethics, ethics training, bioethics training, education, education programs, residents, informed consent, medical practice, ethics competences, bioethics competences, effectiveness and impact.

### Data collection and analysis

After initial searches (February to June 2019), two of the authors (FM, PT) met regularly to discuss potentially relevant articles and whether these met inclusion criteria. Three authors (FM, PT, CL) critically and independently appraised all the reports that met inclusion criteria. The data were extracted using the EndNote software and analysed in Excel spreadsheets.

## Results

This systematic review included 26 studies on ethical medical education programs with quantitative measures: 12 (46%) with medical students, 12 (46%) with residents, 2 (8%) with faculty physicians (see Supplementary File 1 - description of included studies).

**Figure 1.**
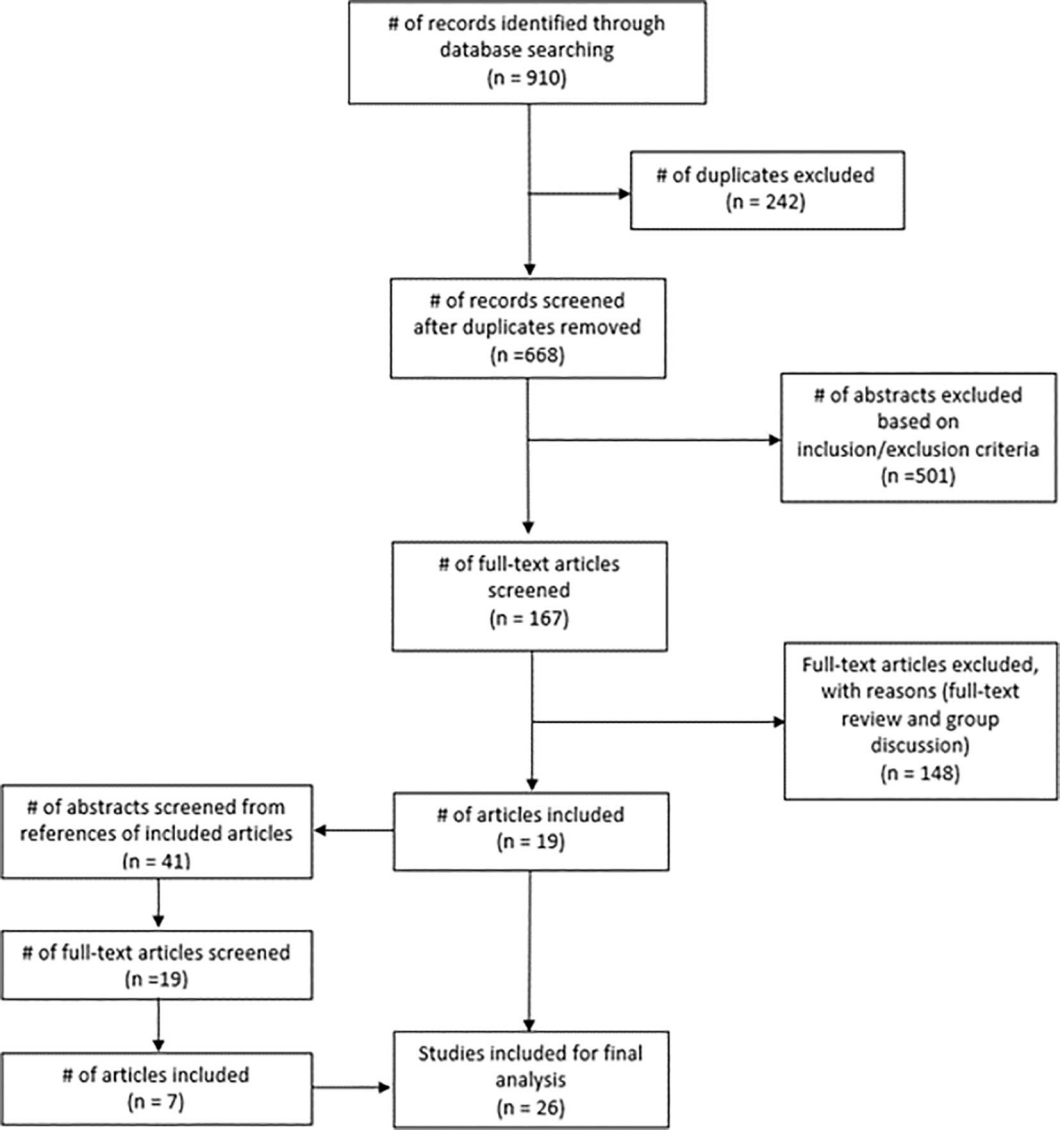
Study flow diagram

Publication years range from 1990 to 2017; twenty of which report studies developed in the United States of America and six that took place in Scotland, England, Canada, New Zealand, Singapore and South Africa.

Studies differ in design: 21 with pre and post intervention evaluation with all participants receiving ethical medical education, 11 with a control group that didn’t receive the intervention. Randomization is only described in 5 studies and 8 reported follow up measures’ months or years after the intervention.

Only one study included pre and post intervention evaluation with control group and follow up ( Sulmasy *et al.,* 1994).

Merely 6 studies combine pre and post intervention evaluation with follow-up (( Self and Olivarez, 1996), ( Goldie *et al.,* 2002), ( Goldie *et al.,* 2003), ( Goldie *et al.,* 2004), ( Hochberg *et al.,* 2012) and ( Didwania *et al.,* 2017)); 1 study combined control group vs experimental group evaluation with follow-up ( Robb *et al.,* 2005); 5 studies combine pre and post intervention evaluation of the experimental group with a control group (( Berseth and Durand, 1990), ( Wenger, Liu and Lieberman, 1998), ( Goldie *et al.,* 2001), ( Chin *et al.,* 2011), ( von Gunten *et al.,* 2017)); comparison between control group and experimental group is present in 4 studies (( Sulmasy *et al.,* 1993), ( Hennen, Morrissy and Grindrod, 1994), ( Coverdale and Turbott, 1997), ( London and McCarthy, 1998)) and 9 studies have only pre and post intervention evaluation (( Shorr, Hayes and Finnerty, 1994), ( Angelos *et al.,* 1999), ( Smith *et al.,* 2004), ( Kitzes *et al.,* 2009), ( Thirunavukarasu *et al.,* 2010), ( Hultman *et al.,* 2012), ( Arora, 2014), ( Lu *et al.,* 2014), ( Kung *et al.,* 2015)). There is a large heterogeneity in ethical medical education programs as regards the contents taught but also the way of teaching.

In order to simplify the studies analysis, intervention was classified in 3 types: type I (i.e. traditional seminar - grand lectures), type II (i.e. case based - small group discussion), type III (i.e. role-play - standardized patients/students).

12 studies have only one type of intervention: nine - type II (( Hennen, Morrissy and Grindrod, 1994), ( Coverdale and Turbott, 1997), ( London and McCarthy, 1998), ( Goldie *et al.,* 2001), ( Smith *et al.,* 2004), ( Kitzes *et al.,* 2009), ( Thirunavukarasu, *et al.,* 2010), ( Kung *et al.,* 2015), ( Didwania *et al.,* 2017)), two - type III (( Lu *et al.,* 2014), ( von Gunten *et al.,* 2017)) and one -type I ( Hochberg *et al.,* 2012) and 14 combine two types (twelve combine type I and II - ( Berseth and Durand, 1990), ( Sulmasy *et al.,* 1993), ( Sulmasy *et al.,* 1994), ( Shorr, Hayes and Finnerty, 1994), ( Self and Olivarez, 1996), ( Wenger, Liu and Lieberman, 1998), ( Goldie *et al.,* 2002), ( Goldie *et al.,* 2003), ( Goldie *et al.,* 2004), ( Smith *et al.,* 2004), ( Chin *et al.,* 2011), ( Hultman *et al.,* 2012), ( Arora, 2014)); two combine type II and III - ( Angelos *et al.,* 1999), ( Robb *et al.,* 2005).

Studies differ in assessment, not only in the outcome measures, but also in the instruments of evaluation. The most common outcomes are Knowledge, Confidence and Attitudes/ Behaviour. The majority of studies evaluate more than one outcome: 2 studies evaluate Knowledge, Confidence and Attitudes/Behaviour - ( Sulmasy *et al.,* 1993), ( Sulmasy *et al.,* 1994); 8 studies evaluate Knowledge and Attitudes/Behaviour - ( Shorr, Hayes and Finnerty, 1994), ( Hennen, Morrissy and Grindrod, 1994), ( London and McCarthy, 1998), ( Goldie *et al.,* 2001), ( Goldie *et al.,* 2002), ( Goldie *et al.,* 2004), ( Robb *et al.,* 2005), ( Hultman *et al.,* 2012); 6 studies evaluate Knowledge and Confidence - ( Angelos *et al.,* 1999), ( Kitzes *et al.,* 2009), ( Thirunavukarasu *et al.,* 2010), ( Chin *et al.,* 2011), ( Arora, 2014), ( von Gunten *et al.,* 2017); 2 studies evaluate Confidence and Attitudes/Behaviour - ( Hochberg *et al.,* 2012), ( Lu *et al.,* 2014).

Eight studies evaluate only one outcome: 2 Knowledge (( Wenger, Liu and Lieberman, 1998), ( Smith *et al.,* 2004)), 6 Attitudes/Behaviour (( Berseth and Durand, 1990), ( Self and Olivarez, 1996), ( Coverdale and Turbott, 1997), ( Goldie *et al.,* 2003), ( Kung *et al.,* 2015), ( Didwania *et al.,* 2017)).

Assessment instruments included knowledge tests ( *i.e.* multiple choice, open-ended question, true-false), self-assessment questionnaires ( *i.e.* likert type scales), reviewing clinical charts and OSCE. Two studies used OSCE in their evaluation (( Robb *et al.,* 2005), ( Hochberg *et al.,* 2012)), 13 studies used knowledge tests, but the majority (21) used surveys/questionnaires in their intervention evaluation. However only 7 studies of those used standardized and validated questionnaires ( *Rest’s defining issue Test*- ( Self and Olivarez, 1996); *Ethics and Health Care Survey instrument* - ( Goldie *et al.,* 2001), ( Goldie *et al.,* 2002), ( Goldie *et al.,* 2003), ( Goldie *et al.,* 2004); *Death Rounds Questionnaire* - ( Kitzes *et al.,* 2009); *Penn State College of Medicine Professionalism Questionnaire* - ( Kung *et al.,* 2015)), the remaining studies developed their own questionnaires/surveys.

Statistically significant differences (positive impact) were found in confidence, attitudes, knowledge in 19 studies. However, in one of these studies ( Berseth and Durand, 1990) the result was statistically significant in one of the six scales use to measure the outcomes. In two studies (( Goldie *et al.,* 2002), ( Goldie *et al.,* 2004)) the positive effect of intervention refers to pilot trial rather than follow-up result and in another study ( Arora, 2014) authors refer a dramatic improvement but statistical information is not provided.

Four studies did not find statistically significant differences (( Shorr, Hayes and Finnerty, 1994), ( Hennen, Morrissy and Grindrod, 1994), ( Coverdale and Turbott, 1997), ( Goldie *et al.,* 2003)). The 4 of them measured Attitudes/Behaviour and two of them measure also Knowledge. The 4 of them receive type II intervention and two of them receive also type I.

3 studies have mixed results (statistically significant differences in one outcome rather than the other) - ( London and McCarthy, 1998), ( Angelos *et al.,* 1999) and ( Hochberg *et al.,* 2012).

With these results, it was not possible to identify an association between a positive impact of ethics medical education with a particuar type of intervention ( *i.e* Type I, II or III) or with an outcome ( *i.e.* knowledge, confidence or attitudes/behaviour). This review contains 8 studies with follow-up, with variable duration, from a few months to a few years. From these trials, 4 maintained positive effect of ethics intervention at the re-assessment at the end of follow-up (( Sulmasy *et al.,* 1994), ( Self and Olivarez, 1996), ( Robb *et al.,* 2005), ( Didwania *et al.* *,* 2017)), 3 didn’t (( Goldie *et al.,* 2002), ( Goldie *et al.,* 2003), ( Goldie *et al.,* 2004)) and one presented mixed results in different outcome measures ( Hochberg *et al* *.,* 2012).

The heterogeneity of included studies in terms of type of training programs, study population, outcomes, measurement instruments and reported statistics limits the possibility of a meta-analysis.

### Risk of bias

The included studies were assessed for risk of biases (see Supplementary File 2). The most frequent was selection bias with very few studies reporting randomization or allocation procedures. Probably because conducting educational randomized control trials requires significant resources (e.g. time, funding or sample size) which is still quite challenging in medical education research.

As most participants in the several studies were a convenience and volunteer sample, this may change the effects of the intervention, as they will tend to be more interested in this topic, perhaps more prepared and possibly different from the study population.

Performance and perception bias were also identified in many studies, participants may have responded or acted based on expectations, what is ethically and morally accepted. The fact that most of the studies do not have blindness could influence the interpretation of some outcome’s measures, especially the subjective ones.

Attrition bias was additionally identified; dropouts without justification may influence results interpretations, because it is very different to dropout to lack of usefulness or the impossibility to continue ( *e.g.* work, health or family).

Some studies have selective reporting of pre-specified outcome like including only a subset of the analysed data or failing to report data that was analysed in the trials, which can potentially compromised validity of the study. Finally, in this review the confounding bias was repeatedly identified. Frequently, it is not possible to explain if the results are due to the normal growing of students/residents over the course/residency, the environment around them, the hidden curriculum or the consequence of medical ethics education.

## Discussion

The studies included in the present review were vastly heterogeneous regarding studies’ design, levels of training of participants, teaching topics and intervention type, assessment methods and outcome measures. Less than half have a comparison with a control group (11) and a minority have follow-up (8). To improve the quality of the findings in the future trials about intervention effectiveness should include beside pre and post intervention, a comparison with a control group and follow-up measurements.

Educational levels of the trainees involved in these studies ranged from first year medical students to faculty physicians. Only 2 studies focus the medical ethics education on post-graduated faculty physician. The disconnection between what medical students/residents are taught about ethical behaviours/attitudes and what is demonstrated by faculty has been identified as one of the main challenges. The development of professional identities and practices is modulated by role models in medicine. Furthermore, the concept of a hidden curriculum although not new is often underestimated. While positive role models reinforce the character and values of the profession, the negative ones may directly contradict classroom lessons and expectations of patients, society and doctors ( Lehmann, Sulmasy and Desai, 2018).

Simulation is a valuable tool that has been applied to medical training and competency-based assessment, in this review was present in only 4 studies. This type of teaching intervention has the advantage of eliciting authentic behaviours under naturalistic conditions that otherwise would be difficult to observe and record systematically ( Wali *et al.,* 2016). The development of OSTEs (Objective Structured Teaching Exercises) have the potential to add value and fill a flaw in medical ethics training and evaluation ( Wali *et al.,* 2016).

Only a few studies used standardised and validated instruments and most of the outcome measures were developed for the particular study and also reporting of the instruments often lacked clarity. Without valid and consistently applied assessment tools the evaluation of ethics education becomes extremely difficult ( Campbell, Chin and Voo, 2007) ( De La Garza *et al.,* 2017).

Outcomes also varied as trials focused on different aspects, mainly knowledge, confidence and attitudes/behaviour. Most trials (19) were able to demonstrate a significant difference before and after their intervention (positive impact on effectiveness of ethics medical education) but some showed no significant difference (4) or mixed results (3).

It was not possible to identify an association between a positive impact of ethics medical education with a particular type of intervention ( *i.e.* type I, II or III) or outcome ( *i.e.* knowledge, confidence or attitudes/behaviour). Most positive findings about effectiveness of ethics medical education were measured after interventions and no conclusions can be drawn about long terms effects.

Several studies have suggested that as the students/residents go further up the course/residency, some seem to lose ethical sensitivity rather than gain more of it. This phenomenon may be due to several factors: entering a culture that devalues ethics, lack of leadership or institutional support ( Campbell, Chin and Voo, 2007) ( Scott *et al.,* 2003).

Teaching is undoubtedly a fundamental value in medicine and learning environment should foster respect, honesty, empower individual concerns about ethics, professionalism and care delivery. Although the importance of patient safety and dignity initiatives are taught in the classroom the most compelling lessons occur in clinical environments. It’s important to educate and train students and residents to deal with ethical issues but not less relevant to teach their teachers how to convey the lesson and to keep their role-models up to date with current ethical concerns. However, health care environment constrains don’t facilitate physicians to have the time required to teach and to become competent role models.

Constrains such as unsupportive leadership, inadequate time with patients, bureaucratic pressures, business climate and non-facilitative practice structures declines the humanistic care and lead to stress, frustrations, dissatisfaction and burnout. All these factors can contribute to a process of loss of effectiveness of ethics medical education throughout time ( Rider *et al* *.,* 2018) ( Shanafelt *et al* *.,* 2015).

Efforts to counteract all these negative consequences have largely focused on supporting and cultivating humanistic attributes in individuals but changes at the organizational level are at least equally important ( Rider *et al* *.,* 2018) ( Montgomery *et al.,* 2013) ( Branch *et al.,* 2001) ( Shanafelt *et al* *.,* 2015). Without positive organisational changes actions focusing only on individuals are unlikely to achieve the purpose of optimal patient care and persistent ethical doctors ( Rider *et al* *.,* 2018) ( Shanafelt *et al* *.,* 2015). Healthcare systems should create and sustain a strong ethical culture, encourage discussion on ethical concerns and create psychologically safe environments for professionals and patients ( Lehmann, Sulmasy and Desai, 2018) ( Shanafelt *et al* *.,* 2015) ( Scott *et al.,* 2003). A great expectation is placed on health care organisations to provide adequate resources and fully recognize their role on medical education.

## Conclusions

Efforts have been made to evaluate ethical medical education programs. A great heterogeneity and weaknesses were found in the way of teaching and in the evaluation’s instruments and measured outcomes. This presents a difficulty for the comparison and interpretation of the results. This review suggests that the impact of teaching medical ethics is positive, however, little is known about which features of the interventions that are most effective and its long-term result.

Ethics is a mainstay in medicine and must be treated with the same diligence and rigor as other clinical skills. In order to scientifically demonstrate the impact of teaching medical ethics it is necessary to carry out quality research that supports it.

Future research may benefit from more robust designs for intervention effectiveness including control groups, pre and post intervention and follow-up measurements; more studies focused on action / behaviour and on teaching faculty physicians. The definition of relevant outcomes for this type of intervention and standardised assessment tools should also be of concern and warranted.

## Take Home Messages

• Ethics is a mainstay in medicine and must be treated with the same diligence and rigor as other clinical skills.
• This review suggests that the impact of teaching medical ethics is positive.
• A great heterogeneity and weaknesses were found in the way of teaching and in the evaluation’s instruments and measured outcomes.
• Future research may benefit from more robust designs for intervention effectiveness including control groups, pre and post intervention, follow-up measurements; more studies focus on behaviour and on teaching faculty physician.

## Notes On Contributors

Filipa Carvalho Moreira, MD (Corresponding author)

Filipa Moreira is an ENT doctor, assistant in the Department of Otorhinolaryngology and Head and Neck Surgery, Hospital de Braga. She is also a faculty member of School of Medicine, Minho’s University and a PhD student at Medical Ethics at the same institution. ORCiD: https://orcid.org/0000-0002-9983-3445

Pedro Miguel Teixeira, MSc, Phd

Pedro Teixeira is a Professor at School of Medicine, Minho’s University, researcher at the Department Population Health - Life and Health Sciences Research Institute (ICVS). His areas of interest are health psychology, community psychology, methodology, quantitative and qualitative research, evidence and decision in Health. ORCiD: https://orcid.org/0000-0001-6322-4923

Cecília Leão, Phd, Full professor

Cecília Leão is a full professor at the School of Medicine of the University of Minho, being, presently, an emeritus Professor, a member of the UMinho Ethics Council and the holder of the chair “Cátedra Alumni Medicina - Professor Pinto Machado” devoted to Bioethics and Humanities in Medicine. ORCiD: https://orcid.org/0000-0003-1311-7884

## Appendices

Abbreviations

**OSCEs:** Objective Structured Clinical Examinations

**OSTEs:** Objective Structured Teaching Exercises
