# Supplementary material for: Effectiveness of medical ethics education: a systematic review
Source: MedEdPublish (2016). 2021 Jun 15;10:175. [Version 1] doi: 10.15694/mep.2021.000175.1 (PMC10939587; doi:10.15694/mep.2021.000175.1)
Supplement: Peer review history

# Reviewer report: Reviewer response for version 1

**DOI:** 10.21956/mep.18933.r27077

**Published:** 2021-10-16

**Authors:** Irina Markovina

**Affiliations:** 1 Sechenov First Moscow State Medical University (Sechenov University)

This review has been migrated. The reviewer awarded 3 stars out of 5 I find the theme of the article very important and interesting. The research presented focuses on one of the most difficult issues in medical education, namely, how to evaluate the results of medical ethics teaching and what tools seem to be most effective in both teaching and evaluating its results. We teach medical ethics and medical humanities trying to educate and train very good healthcare professionals. However, to evaluate the results of this training and to decide which of the tools is most effective and informative remains a challenging task; I would not disagree with the authors in that. To me, it looks like a classical educational systematic review. The article is well structured and has all the required components: introduction, objectives, methods, results, discussion, etc. As regards the content of these traditional parts, I would recommend to be clearer about one section, Methods, which in my opinion should have a more appropriate name of Methods and Materials. The research is presented in a detailed way with all the steps of data collection, analysis and discussion. The conclusions are informative and take home messages look like the programme for future research.Another minor recommendation of mine would be to have the article proofread again to remove grammar slips and make the English language better. As a non-native English speaker, I am always concerned about it.My conclusion is the article is definitely worth indexing.

Reviewer Expertise:

NA

No decision status is available

# Reviewer report: Reviewer response for version 1

**DOI:** 10.21956/mep.18933.r27079

**Published:** 2021-10-12

**Authors:** Richard Hays

**Affiliations:** 1 James Cook University

This review has been migrated. The reviewer awarded 4 stars out of 5 I agree with other reviewers that qualitative evaluations may well contribute further, but the impact of ethics education will remain hard to measure because it is subsequent behaviour in practice - often many years later - that is the most important outcome. Knowledge is more easily quantified and so is more commonly used in studies of impact of educational interventions. While improvement in knowledge of ethical principles and ethical decision-making processes is important, does that reduce subsequent lapses in ethical behaviours or foster genuine change in attitudes? This is where the qualitative studies may contribute. Perhaps even more important than knowledge is the role modeling in the workplace and culture of thoughtful discussion and application of ethical principles. This article is a worthwhile contribution to the literature and should encourage ethics teachers and researchers to aim higher.

Reviewer Expertise:

NA

No decision status is available

# Reviewer report: Reviewer response for version 1

**DOI:** 10.21956/mep.18933.r27080

**Published:** 2021-10-12

**Authors:** Ken Masters

**Affiliations:** 1 Sultan Qaboos University

This review has been migrated. The reviewer awarded 3 stars out of 5 An interesting systematic review of the effectiveness of medical ethics education.The subject is important, as medical ethics is frequently taught, but the impact of such teaching is also frequently questioned when individual cases of unethical behaviour among health professionals occur.The study has been well-conducted, and the authors have presented their work in a clear and organised way. The detailed analysis of potential bias (Supplementary file 2) is appreciated (this aspect is often ignored in reviews, or glossed over in a sentence or two, so it is good to see this performed properly).Major point:• A very real problem exists in the unexplained exclusion of qualitative studies. This exclusion is particularly surprising because, in their Introduction, the authors acknowledge that “Qualitative methods will capture a more holistic perspective and have the potential to present a better understanding into how and why ethics education is effective”. So, given that the authors are aware of qualitative studies’ strengths, the exclusion of qualitative studies is strange, and severely undermines that paper. We do not know how many of these were found and removed (the box shows merely that 501 were excluded because of the exclusion criteria). As a result, the concluding remark that “little is known about which features of the interventions that are most effective and its long-term result” should be viewed with some scepticism, because it is possible that authors have excluded crucial studies from their review.Minor point• Figure 1 can be confusing, as it shows two arrows going from the box labelled “# of articles included (n=19)". I suggest that it should have the vertical arrow only, and the box to the left should be the start of a new thread of boxes and arrows.So, the paper is mostly well-written, but the exclusion of qualitative studies severely undermines its value, and the conclusions (particularly as they are expressed in the Abstract) cannot be taken at face value. Given the amount of work that the authors have put into this research, this is, indeed, a great pity.

Reviewer Expertise:

NA

No decision status is available

# Reviewer report: Reviewer response for version 1

**DOI:** 10.21956/mep.18933.r27078

**Published:** 2021-06-18

**Authors:** Roger Worthington

This review has been migrated. The reviewer awarded 3 stars out of 5 This is a timely piece of work, and much effort has been expended in collating and analyzing the information. The paper addresses issues that often come up in meetings about curriculum development. Medical educators might like numbers to show that an intervention works, but ethics is not an exact science, and by the same token, the effectiveness of teaching ethics cannot be easily assessed. These are longstanding issues that medical schools and program directors have to deal with, and while some ‘facts’ under the heading ‘medical ethics’ can be assessed and measured, this typically tests the ability to retain information more than levels of understanding. In my view, the value in teaching medical ethics lies in its ability to influence future professional behaviors and decision-making. 'Soft skills’ can indeed be assessed using a mix of OSCEs, short answer questions and case study analysis, and while it may be expedient to use multiple choice and Likert scale questionnaires to assess the knowledge of large numbers of medical students in their first or second years, such methods are less-well suited to clinical years and post-qualification assessment. Only two of the studies included in this survey used OSCEs in their evaluation, and the decision to “exclude studies with qualitative outcome measures and opinion or revision papers” [p.13] is puzzling, since qualitative studies might have yielded quality results. Someone well trained in ethics should be equipped to navigate the muddy waters of clinical practice and know how to reach a critical decision, which often means choosing between shades of grey rather than between black and white. Therefore, if “the primary objective is to analyze the impact of training in medical ethics, and secondly to describe educational practices and discuss the most effective and appropriate pedagogical models [p.11], this might mean adopting a different methodology.

Reviewer Expertise:

NA

No decision status is available
